# The collection and database of Birds of Angola hosted at IICT (Instituto de Investigação Científica Tropical), Lisboa, Portugal

**DOI:** 10.3897/zookeys.387.6412

**Published:** 2014-03-11

**Authors:** Miguel Monteiro, Luís Reino, Pedro Beja, Michael Stuart Lyne Mills, Cristiane Bastos-Silveira, Manuela Ramos, Diana Rodrigues, Isabel Queirós Neves, Susana Consciência, Rui Figueira

**Affiliations:** 1 Instituto de Investigação Científica Tropical, R. da Junqueira, 86 - 1°, 1300-344, Lisboa, Portugal; 2 CIBIO, Centro de Investigação em Biodiversidade e Recursos Genéticos / InBIO, Universidade do Porto, Vairão, Portugal; 3 Percy FitzPatrick Institute of African Ornithology, DST/NRF Centre of Excellence, University of Cape Town, South Africa; 4 A. P. Leventis Ornithological Research Institute, University of Jos, PO Box 13404, Jos, Plateau State, Nigeria; 5 CESAM-Centre for Environmental and Marine Studies, Universidade de Aveiro, Aveiro, Portugal; 6 Museu Nacional de História Natural e da Ciência, Universidade de Lisboa, Portugal

**Keywords:** Occurrence, Specimen, Angola, Instituto de Investigação Científica Tropical (IICT), Animalia, Chordata, Aves

## Abstract

The bird collection of the Instituto de Investigação Cientítica Tropical (Lisbon, Portugal) holds 5598 preserved specimens (skins), mainly from Angola, Mozambique, Guinea-Bissau, São Tomé and Principe, and Cape Verde. The subset collection from Angola includes 1560 specimens, which were taxonomically revised and georeferenced for the publication of this data paper. The collection contains a total of 522 taxa, including 161 species and 361 subspecies. Two species are classified by the IUCN Red List as Endangered - the wattled crane (*Grus carunculata*) and the Gabela bush-shrike (*Laniarius amboimensis*) - and two are classified as vulnerable - African penguin (*Spheniscus demersus*) and the white-headed vulture (*Trigonoceps occipitalis*). The temporal span of the database ranges between 1943 and 1979, but 32% are from years 1958–1959, and 25% from years 1968–1969. The spatial coverage of the collection is uneven, with 2/3 of the records representing only four of the eighteen provinces of the country, namely Huíla, Moxico, Namibe and Cuanza Sul. It adds, however, valuable information for the Huíla area of the Angolan Scarp, which is probably a biodiversity hotspot of global conservation priority. Furthermore, this georeferenced database adds invaluable bird information to the GBIF network, for one of the countries with highest but less known biodiversity in Africa.

## Introduction

Angola is one of the countries in Africa with highest bird diversity (938 native species, according to Mills and Melo (2013)), including a high number of endemic and threatened species (Stattersfield et al. 1998). It encompasses four main types of ecosystems: Congo lowland basin forests in the north, Angolan miombo woodlands in the centre, Zambesian miombo woodlands in the east, and Namib Desert in the south-west (Dean 2000). Despite its richness, Angola is still one of the least known countries for birds. This lack of knowledge is mainly a consequence of both the Portuguese Colonial war (1961–1974) and the Angolan civil war (1974–2002), which together lasted 41 years (1961–2002), halting scientific studies and expeditions (Dean 2000). Since the end of the civil war, Angolan society and government have focused primarily on infrastructure reconstruction and economic development, with limited attention given to scientific research and natural history studies.

Even today, basic information on Angolan bird species dates mostly from before the national independence in 1974 (Dean 2000, Ministry of Environment 2009). Some recent work has updated our knowledge to some degree (Ryan et al. 2004, Mills 2009, 2010, Mills and Dean 2007, Mills et al. 2011, 2013), including the publication of a national check-list (Mills and Melo 2013). However, historical collections still play a major role in the description of the country’s biodiversity. Access to the substantial information collected on the Angolan avifauna is of great importance, considering that the IUCN Red List indicates, the occurrence in the country of one critically endangered, 14 endangered and 10 vulnerable bird species (IUCN 2013). Records should thus be made available in a form that can be readily found and used.

In this paper we provide a comprehensive dataset based on the digitalization, taxonomic revision and georeferencing of the Angolan ornithological collection held by the Instituto de Investigação Científica Tropical (IICT), Lisbon. The dataset is freely available via the Internet, on the IICT IPT provider (http://maerua.iict.pt/ipt), and on the Global Biodiversity Information Facility (GBIF) data portal (http://www.gbif.org). It comprises information on 1560 specimens collected in 291 localities throughout Angola. The specimens were collected in expeditions carried out between 1949 and 1979, by 64 collectors. The collection contains some very valuable skins of endemic species, such as of the endemic Red-crested Turaco (*Tauraco erythrolophus* (Vieillot, 1819)) and Grey-striped Francolin (*Pternistis griseostriatus* (Ogilvie-Grant, 1890)). It also contains skins of species listed as conservation concern in IUCN Red List, including two endangered species (the wattled crane (*Grus carunculata* (Gmelin, JF, 1789)) and the Gabela bush-shrike (*Laniarius amboimensis* Moltoni, 1932)), of which there are few skins in other collections (Dean 2000). There are also two species classified as vulnerable (African penguin (*Spheniscus demersus* (Linnaeus, 1758)) and the white-headed vulture (*Trigonoceps occipitalis* (Burchell, 1824))).

## General description

The dataset is a subset of the parent bird collection of the Instituto de Investigação Cientítica Tropical, which holds 5598 preserved specimens (skins), mainly from Angola, Mozambique, Guinea-Bissau, São Tomé and Principe, and Cape Verde, available through GBIF at http://maerua.iict.pt/ipt/resource.do?r=iict_cz. The collection scrutinized through this data paper is the subset from Angola, which includes 1560 specimens that were taxonomically revised and georeferenced. The collection shares the largest collectors (A. Rosa Pinto, D. Mumputu and J. Carlos) with the related biggest collection of birds of Angola, based on Instituto Superior de Ciências de Educação da Huíla (ISCED-Huíla), in Lubango. That institute inherited the collections of the former Instituto de Investigação Científica de Angola (IICA), including a bird collection with more than 35 thousand specimens, making it the largest in Africa. Although showing an uneven geographic distributions of samples, with 2/3 of the records concentrated in only four provinces (Huíla, Moxico, Namibe and Cuanza Sul), the collection adds, invaluable information for the Huíla’s area of the Angolan Scarp, which is probably a biodiversity hotspot of global conservation priority (Myers et al. 2000), and an important area of bird endemism (Mills 2010).

## Project details

**Project title:** Online Catalogue of Biological Collections of IICT

**Funding:** This project was funded by the Fundação para a Ciência e a Tecnologia (FCT) through the project “Recovering the past, recording the present, and preparing the future of zoological collections in Portugal (ARCA)” (PTDC/BIA-QOR/71492/2006) and co-funded by CIBIO, Centro de Investigação em Biodiversidade e Recursos Genéticos / InBIO from the University of Porto.

## Taxonomic coverage

**Figure 1.**
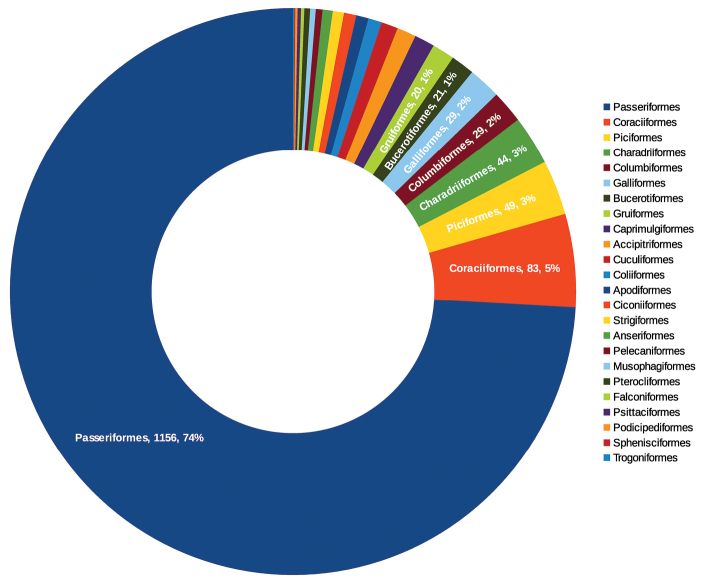
Number and percentage of specimens per orders. Only the categories of orders having 20 or more specimens are labeled.

**Figure 2.**
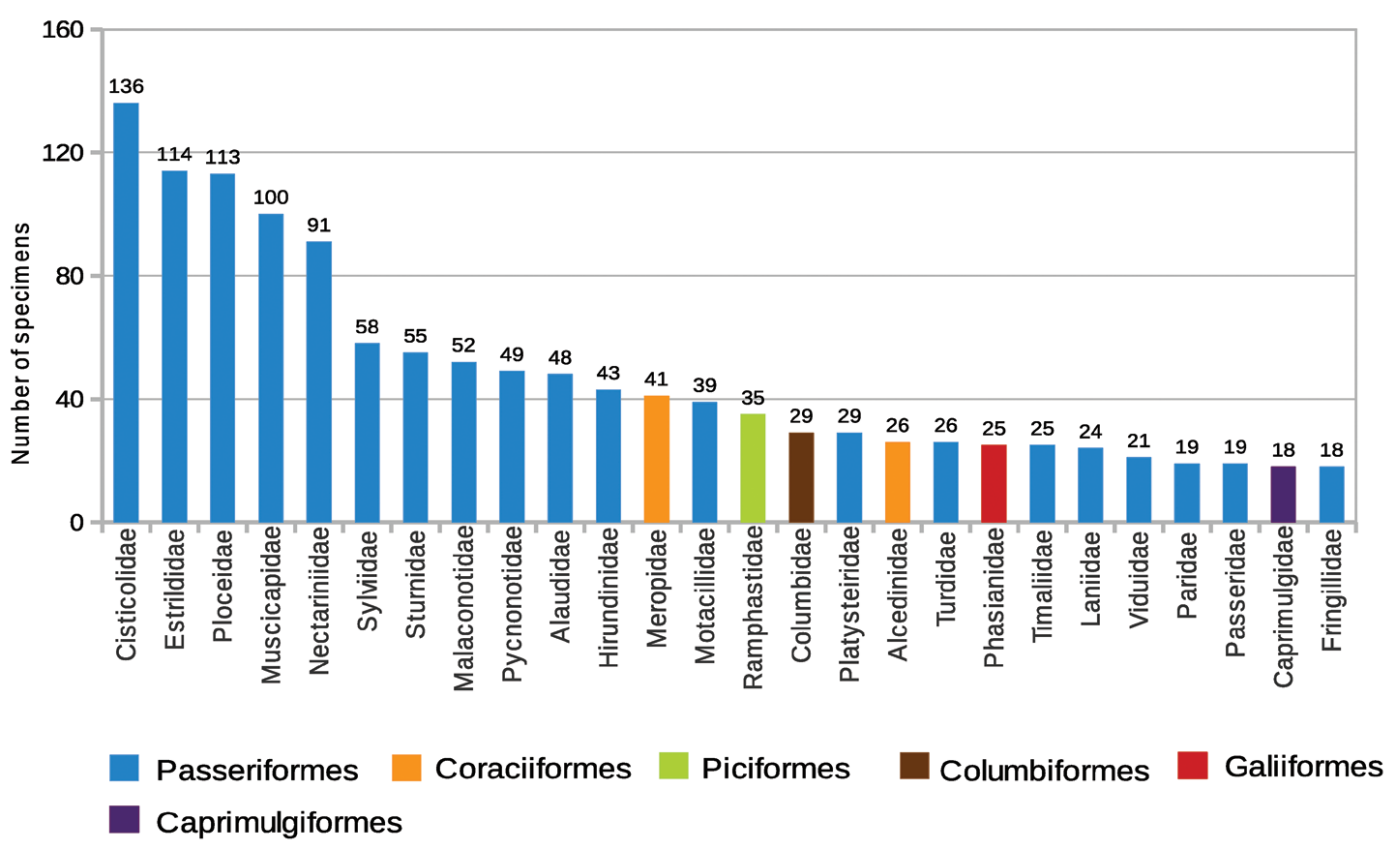
Number of specimens per family. The families pictured represent 80% of the number of specimens in the collection.

**General taxonomic coverage description:** The taxonomic coverage of this dataset spans class, and it includes 24 orders and 69 families (Figure 1). Nearly two thirds of the specimens belong to the order Passeriformes. The Coraciiformes order ranks second, with 5% of the specimens. The families Cisticolidae, Estrildidae and Ploceidae have the highest number of records (136, 114 and 113 records respectively) (Figure 2). The families with fewest records are Bucorvidae, Ciconiidae, Picidae, Spheniscidae, Trogonidae, Turnicidae and Tytonidae, with one record each. The database contains 522 taxa (161 species and 361 subspecies).

## Taxonomic ranks

**Kingdom:** Animalia

**Phylum:** Chordata

**Class:** Aves

**Order:** Accipitriformes, Anseriformes, Apodiformes, Bucerotiformes, Caprimulgiformes, Charadriiformes, Ciconiiformes, Coliiformes, Columbiformes, Coraciiformes, Cuculiformes, Falconiformes, Galliformes, Gruiformes, Musophagiformes, Passeriformes, Pelecaniformes, Piciformes, Podicipediformes, Psittaciformes, Pterocliformes, Sphenisciformes, Strigiformes, Trogoniformes

**Family:** Accipitridae, Alaudidae, Alcedinidae, Anatidae, Apodidae, Ardeidae, Bucerotidae, Bucorvidae, Campephagidae, Caprimulgidae, Certhiidae, Charadriidae, Ciconiidae, Cisticolidae, Coliidae, Columbidae, Coraciidae, Cuculidae, Dicruridae, Emberizidae, Estrildidae, Eurylaimidae, Falconidae, Fringillidae, Glareolidae, Gruidae, Hirundinidae, Indicatoridae, Jacanidae, Laniidae, Laridae, Malaconotidae, Meropidae, Monarchidae, Motacillidae, Muscicapidae, Musophagidae, Nectariniidae, Numididae, Oriolidae, Otididae, Paridae, Passeridae, Phalacroracidae, Phasianidae, Phoeniculidae, Picidae, Platysteiridae, Ploceidae, Podicipedidae, Psittacidae, Pteroclidae, Pycnonotidae, Rallidae, Ramphastidae, Remizidae, Scolopacidae, Spheniscidae, Strigidae, Sturnidae, Sylviidae, Timaliidae, Trogonidae, Turdidae, Turnicidae, Tytonidae, Upupidae, Viduidae, Zosteropidae

**Common names:** Birds

## Spatial coverage

**Figure 3.**
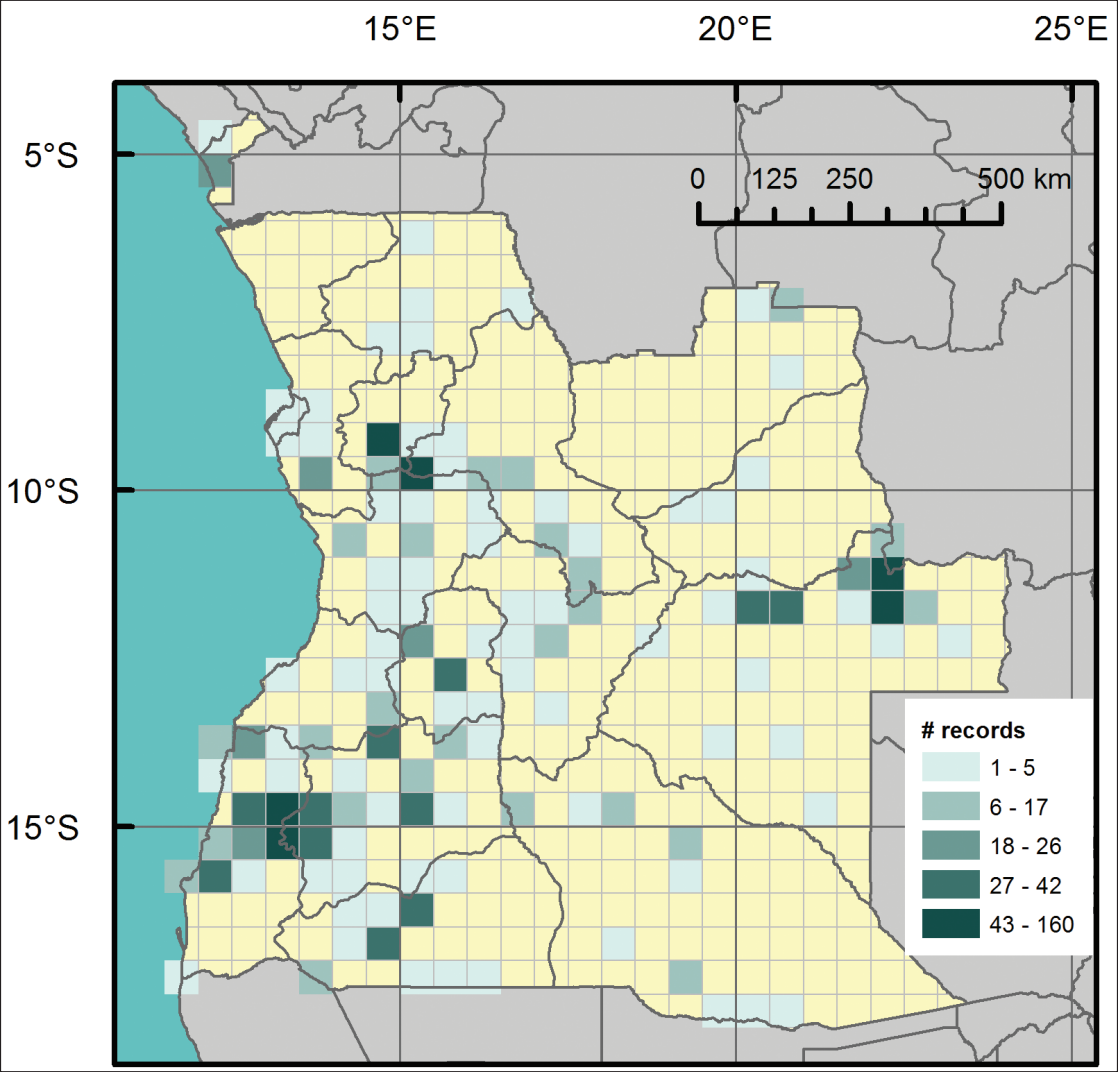
Distribution of occurrence records with indication of number of records indicated on a half a minute grid system.

**General spatial coverage:** The geographic range of the collection covers the whole Angola. Distribution of sampling locations is presented in Figure 3, including counts of records per grid cell, in a half a minute grid. The distribution among the Angolan provinces is uneven, with the following series: Huíla (320), Moxico (293), Namibe (202), Cuanza Sul (166), Cuanza Norte (107), Cunene (88), Cuando Cubango (54), Huambo (51), Malanje (51), Benguela (35), Bengo (29), Cabinda (26), Bie (21), Lunda Norte (16), Lunda Sul (9), Uige (9), Luanda (1). No records occur in the province of Zaire, in the north-west region of Angola. The province of collection is unknown for 82 specimens. The research unit in Angola where the main collectors where based was located in Huíla, which justifies the highest value found for that province.

### Coordinates

18°30'36"S and 4°5'60"S Latitude; 10°2'24"E and 24°51'0"E Longitude

### Temporal coverage

**Figure 4.**
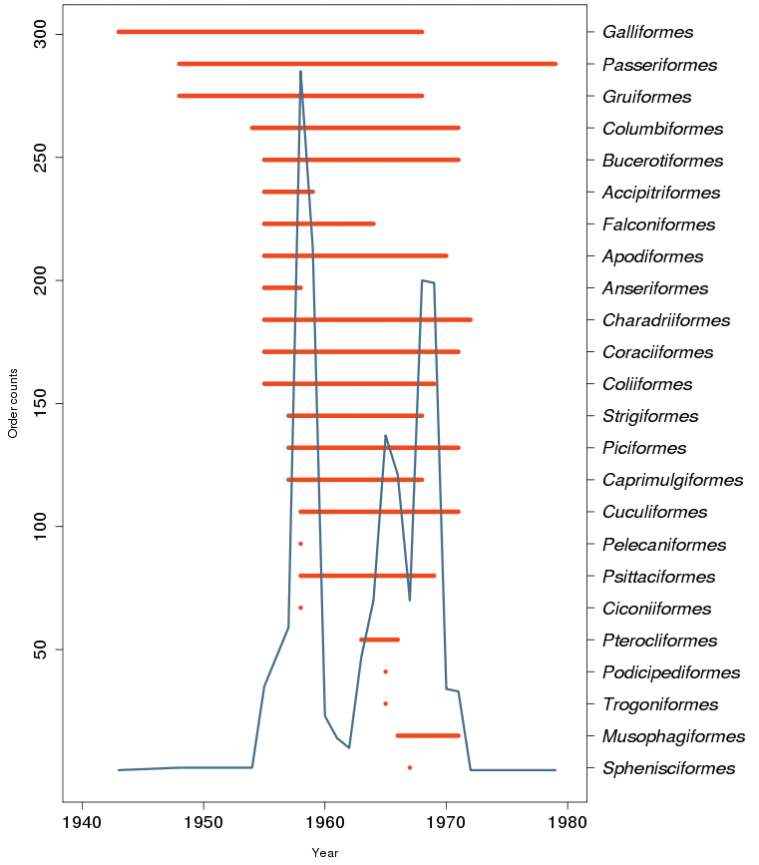
Temporal profile of the specimens in the collection. The time range for each order is represented by the horizontal bars.

The temporal range of the records is between 1943 and 1979, (Figure 4). Two peak periods are observed, in 1958–1959, and in 1968–1968, with more than 200 samples per year.

## Natural collections description

**Parent collection identifier:** 24798813-aaff-4292-98ef-8c9bc415ff14

**Collection name:** IICT - Colecção de Aves de Angola

**Collection identifier:** 9B48F857-91B6-4029-9AEF-A80F7852EC89

**Specimen preservation method:** Dried

**Curatorial unit:** 1560 with an uncertainty of 0 (skins)

## Methods

**Figure 5.**
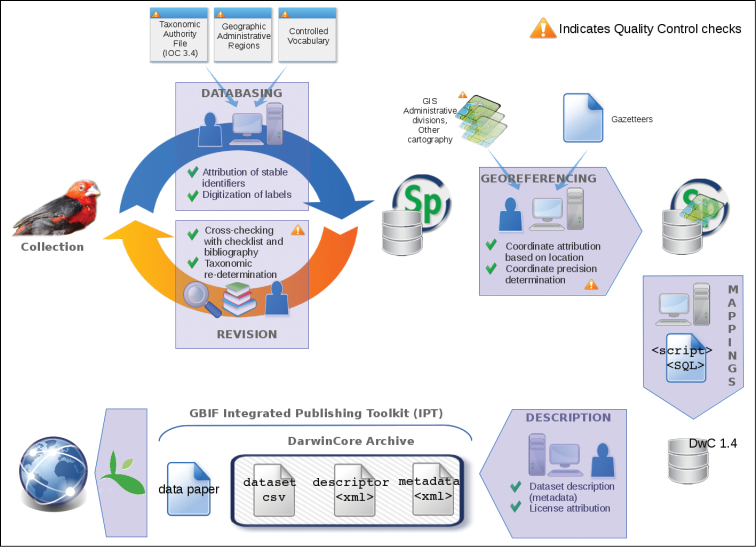
Synoptic of the procedure to digitize and publish the dataset online.

**Method step description:** The general procedure for the processing of specimens databasing and georeferencing is represented in Figure 5. The mammal and bird collections of the IICT were initially catalogued under the scope of project ARCA (2008–2010), using the software Specify Workbench, and afterwards imported to software Specify version 6 (Specify Software Project 2013). Whenever available, the descriptions of eye, beak and foot colour, and total length were also included.

Since at that time no taxonomic specialists were available to revise the collection, records were catalogued as they were labelled, without taxonomic revision or update of taxonomic names.

In 2012–2013, the IICT collection of Angolan birds was fully taxonomically revised. This taxonomic revision followed the IOC bird list (Gill and Donsker 2013) and all skins were labelled with a new catalogue number and the original information was thereafter included in the collection manager software Specify 6. Additionally, the specimens’ original information was re-checked at this phase for any initial cataloguing errors.

Since no georeferencing information was available on specimen labels or associated documentation, geographic coordinates were determined following procedure and recommendations by Chapman and Wieczorek (2006). Several geographic gazetteers were used to determine coordinates, based on the location information: Geolocate, Google Maps, Google Earth. Furthermore, the cartographic series 1:100 000 of Angola was used to find additional sites not available at gazetteers or to fine-tune coordinate uncertainty. The uncertainty of the coordinate was recorded whenever possible. For 88 records it was not possible to determine coordinates due to insufficient location information. All coordinates are given in geographic format, decimal degrees, datum WGS 84.

**Study extent description:** The study covers most of Angola, including 17 out of the 18 provinces. The best-represented provinces are Huíla, Moxico, Namibe and Cuanza Norte. Only the province of Zaire (NW Angola) is not represented in the collection. The temporal distribution is mainly concentrated in the decades of 1950s (especially in years 1958 and 1959) and 1960s, corresponding to 95% of the records.

**Sampling description:** More than one thousand records of this dataset resulted from expeditions and studies carried out by the former Section of Ornithology at the Instituto de Investigação Científica de Angola, coordinated by António Augusto da Rosa Pinto between 1958 and 1974. Some scientific results of these studies, for the non-passerine group were published in Rosa Pinto (1983).

**Quality control description:** Information from each specimen was catalogued in Specify 6, which involved two steps: i) digitalization of specimen’s records (performed by MR, DR, IQN and SC); and ii) taxonomic revision and data checking (performed by the first author). The authors LR and MM also contributed to taxonomic revision of the specimens. Scientific names were checked with a taxonomic thesaurus built from the IOC World Bird List (v 3.34) (Gill and Donkster 2012). Georeferencing followed recommendations by Chapman and Wieczorek (2006), including the determination of uncertainty of coordinates, in particular when no sufficient information was available from the specimens’ records and label, to attribute a specific locality of origin (e.g. names of administrative regions, names of rivers).

## Datasets

### Dataset description

**Object name:** Darwin Core Archive The collection and database of Birds of Angola hosted at IICT (Instituto de Investigação Científica Tropical), Lisboa, Portugal

**Character encoding:** UTF-8

**Format name:** Darwin Core Archive format

**Format version:** 1.0

http://maerua.iict.pt/ipt/archive.do?r=iict_bird_angola

**Distribution:** http://maerua.iict.pt/ipt/archive.do?r=iict_bird_angola

**Publication date of data:** 2013-10-09

**Language:** Portuguese

**Licenses of use:** Use of the data for commercial or for-profit applications is permitted only via written permission from Instituto de Investigação Científica Tropical. Data are provided to users, but should not be passed on to third parties or redistributed. It is explicitly forbidden to incorporate these data into other databases of free or restricted access.

**Metadata language:** English

**Date of metadata creation:** 2013-08-22

**Hierarchy level:** Dataset
